# Association of Insulin Resistance Indexes to Carotid Intima–Media Thickness

**DOI:** 10.1371/journal.pone.0053968

**Published:** 2013-01-09

**Authors:** Angela Sciacqua, Maria Adelaide Marini, Marta Letizia Hribal, Francesco Perticone, Giorgio Sesti

**Affiliations:** 1 Department of Medical and Surgical Sciences, University “Magna Graecia” of Catanzaro, Catanzaro, Italy; 2 Department of Internal Medicine, University of Rome-Tor Vergata, Rome, Italy; University of Bari, Italy

## Abstract

**Objectives:**

Accumulating evidence suggests an association between insulin resistance (IR) and cardiovascular diseases The aim of this study was to examine the relationship between indexes of IR and common carotid intima–media thickness (IMT), an indicator of vascular damage.

**Methods:**

In 847 non-diabetic Caucasians a 75 g oral glucose tolerance test was performed and surrogate indexes of IR were computed according to published formulas. IMT was measured by ultrasound method.

**Results:**

The Stumvoll ISI_OGTT_ index was correlated with IMT more strongly than the other indexes of IR. The IR indexes correlated significantly (*P*<0.0001) with all cardiovascular risk factors examined. The Stumvoll ISI_OGTT_ index was correlated with waist circumference and high sensitivity C-reactive protein more strongly than the other indexes of IR. The area under the ROC curve (AUC), used to evaluate the accuracy of the IR indexes in identifying individuals with vascular damage defined as IMT >0.9 mm, for the Stumvoll ISI_OGTT_ index was significantly higher (0.710) as compared with the AUCs of Matsuda (0.642) (*P* = 0.0009), OGIS (0.666) (*P* = 0.04), HOMA (0.611) (*P*<0.0001) and Liver IR (0.648) (*P* = 0.0008) indexes. In a logistic regression model adjusted for age and gender, subjects in the lowest tertile of the Stumvoll ISI_OGTT_ index had the highest risk of having vascular damage (OR 4.95, 95% CI 2.99–8.192) as compared to the corresponding tertiles of the other surrogate indexes.

**Conclusion:**

The Stumvoll ISI_OGTT_ index correlated more strongly than other validated surrogates indexes of IR with carotid IMT, and, therefore, it might be a significant indicator of vascular damage.

## Introduction

Cardiovascular disease is one of the major causes of mortality and morbidity in adult individuals. Insulin resistance (IR) is considered an important risk factor for both atherosclerotic cardiovascular disease [1], [2] and type 2 diabetes [3], and a key determinant of cardiovascular risk factors, including visceral obesity, atherogenic dyslipidemia, and hypertension, clustering within the metabolic syndrome [4]–[6]. Accumulating evidence suggests an association between hyperinsulinemia and/or IR and cardiovascular diseases [7]–[10]. Importantly, it has been shown that IR, evaluated by the frequently sampled intravenous glucose tolerance test with minimal model analysis [11] or by the euglycemic hyperinsulinemic clamp [12], rather than hyperinsulinemia, was independently associated with early vascular atherosclerosis. However, methods to directly measure IR are complex, time-consuming, expensive, and unsuitable for large scale epidemiological studies. Therefore, surrogate indexes of insulin sensitivity have been developed using fasting insulin and/or glucose levels alone [13] or in combination with insulin and glucose levels during an oral glucose tolerance test (OGTT) [14] as well as with other metabolic variables [15]–[17]. Since IR occurs in multiple tissues, these indexes reflects predominantly either hepatic [13], [17] or muscle IR [14]–[16].

Whether these IR indexes are differentially associated with vascular atherosclerosis is still undefined. Intima–media thickness (IMT) of common carotid artery is a well-recognized index of vascular damage, and is widely utilized as a surrogate marker for cardiovascular disease [18].

The aim of this study was to examine the relationship between different indexes of insulin resistance and common carotid IMT in a cohort of nondiabetic Caucasian individuals.

## Materials and Methods

### Ethics Statement

The Protocol was approved by the Ethical Committee “Comitato Etico Azienda Ospedaliera Universitaria Mater Domini”. Informed written consent was obtained from all participants. All the investigations were performed in accordance with the principles of the Declaration of Helsinki.

### Subjects

847 non-diabetic Caucasian subjects, aged 30–70 years, consecutively recruited in the University of Rome and the University of Catanzaro areas participated to this cross-sectional study for assessment of cardio-metabolic risk factors as previously described [19], [20]. Subjects were excluded if they had history of cardiovascular disease including peripheral atherosclerosis, chronic gastrointestinal diseases associated with malabsorption, chronic pancreatitis, history of any malignant disease, history of alcohol or drug abuse, positivity for antibodies to hepatitis C virus (HCV) or hepatitis B surface antigen (HBsAg), and liver or kidney failure. Subjects were excluded if they had diabetes mellitus, defined as ≥126 mg/dl or 2-h post-challenge plasma glucose ≥200 mg/dl. After a 12-h fasting, all subjects underwent anthropometrical evaluation including assessment of body mass index (BMI), and waist circumference, and readings of clinic blood pressure (BP) obtained in the left arm of the supine patients, after five minutes of quiet rest, with a sphygmomanometer. Values were calculated as the average of the last two of three consecutive measurements obtained at 3-mins intervals. A 75 g oral glucose tolerance test (OGTT) was performed with 0, 30, 60, 90 and 120 min sampling for plasma glucose and insulin determination.

IMT of the common carotid artery was measured by ATL HDI 3000 ultrasound system (Advanced Technology Laboratories, Bothell, WA) equipped with a 5 MHz linear array transducer as previously described [12]. Manual measurements were conducted in plaque-free portions of the 10-mm linear segment proximal to the carotid bulb. For each patient two measurements were performed bilaterally, and the values were averaged, to obtain the mean of IMT of the common carotid artery. Ultrasound studies were performed by an experienced examiner who was unaware of the subjects’ clinical and laboratory findings. A value of IMT >0.9 mm was used as index of vascular damage according to the 2007 Guidelines for the management of arterial hypertension released by the Task Force for the Management of Arterial Hypertension of the European Society of Hypertension (ESH) and of the European Society of Cardiology (ESC) [21].

### Analytical Determinations

Glucose, triglyceride, total and high density lipoprotein (HDL-C) cholesterol concentrations were determined by enzymatic methods (Roche, Basel, Switzerland) and plasma insulin concentration was assessed with a chemiluminescence-based assay (Immulite®, Siemens, Italy). High sensitivity C-reactive protein (hsCRP) levels were measured by an automated instrument (CardioPhase® hsCRP, Siemens, Italy).

### Calculations

The homeostasis model assessment (HOMA) index was calculated as fasting insulin×fasting glucose/22.5 [13]. The Matsuda index was calculated as 10,000/square root of [fasting glucose (mmol/L)×fasting insulin (mU/L)]×[mean glucose×mean insulin during OGTT] [14]; the Matsuda2 index was calculated as 10,000/square root of [fasting glucose (mmol/L)×fasting insulin (mU/L)]×[mean glucose_30–120_×mean insulin_30–120_ during OGTT] not including in the formula fasting glucose and insulin levels in mean values of glucose and insulin during OGTT; the Stumvoll Insulin Sensitivity Index (ISI)_OGTT_ was calculated as 0.226 − 0.0032×BMI − 0.0000645×Insulin_120_ − 0.00375×Glucose_90_ [15]; the oral glucose insulin sensitivity (OGIS) was calculated as previously described using a spreadsheet calculator downloaded from http://webmet.pd.cnr.it/ogis [16]; the liver IR index was calculated using the formula: −0.091+ (log insulin AUC 0–120 min×0.400)+(log fat mass %×0.346) - (log HDL Cholesterol×0.408)+(log BMI×0.435) [17]. The trapezoidal method was used to calculate glucose and insulin AUC during an OGTT.

### Statistical Analysis

Triglycerides values were natural log transformed for statistical analysis due to their skewed distribution. Continuous data are expressed as means ± SD. Comparisons between two groups were performed using unpaired Student’s t-test. Categorical variables were compared by χ^2^ test. Relationships between variables were determined by Pearson’s correlation coefficient (r). Partial correlation coefficients adjusted for age and gender were computed between variables. The ability of each index to detect subjects with vascular damage, defined as IMT >0.9 mm [21], was assessed by the area under the receiver operating characteristic (ROC) curve. The area under the ROC curve (AUC) was used as a measure of how well IR indexes identify vascular damage. An AUC of 1.0 indicates perfect classification of individuals with vascular damage, whereas 0.5 means that the classification is not better than chance. Statistical differences between the AUC under the ROC curves were determined by the method described by DeLong et al. [22]. A multiple regression analysis was performed to estimate the independent contribution of indexes of IR to carotid IMT. A multivariable logistic regression analysis was used to determine the association between vascular damage and the tertiles of IR indexes. A *P* value <0.05 was considered nominally significant. All analyses were performed using SPSS software programme Version 16.0 for Windows.

## Results

Men were older, had more central adiposity, but lower fat mass than women (Table 1). Men also had higher BP, fasting, 90 min, and 2-h post-load plasma glucose concentration, fasting insulin levels, triglycerides, and carotid IMT, and lower HDL cholesterol. Men were more insulin resistant than women as they showed significantly higher values of HOMA and Liver IR indexes and lower values of Matsuda, Matsuda2 and OGIS indexes (Table 1). By contrast, no gender-dependent differences in Stumvoll ISI_OGTT_ values were observed probably due to the lack of differences between man and women in two variables considered in the index calculation i.e. BMI and Insulin_120_.

**Table 1:** Anthropometric and metabolic characteristics of the study subjects.

Variables	Whole study group	Men	Women	*P*
Gender (M/F)	414/433	414	433	0.079
Age *(yrs)*	48.4±10.0	49.1±10.0	47.7±9.9	0.04
BMI *(kg/m^2^)*	29.6±5.9	29.4±4.8	29.9±6.7	0.24
Waist circumference (c*m*)	98±13	101±11	96±14	<0.0001
Fat mass (*%*)	32.7±9.9	27.6±7.7	37.6±9.3	<0.0001
SBP *(mmHg)*	131±16	134±15	127±17	<0.0001
DBP *(mmHg)*	82±10	84±10	79±10	<0.0001
Fasting Glucose (*mgl/dl*)	94±12	97±11	92±11	<0.0001
90 min Glucose (*mgl/dl*)	140±43	147±44	134±41	<0.0001
2-h Glucose (*mgl/dl*)	124±34	127±35	121±33	0.003
Fasting Insulin *(µU/ml)*	13±8	13±9	12±7	0.02
2-h Insulin *(µU/ml)*	86±77	90±75	82±78	0.16
Total cholesterol (*mg/dl*)	203±36	203±35	203±37	0.97
HDL (*mg/dl*)	50±13	44±10	56±13	<0.0001
Triglycerides (*mg/dl*)	133±85	152±98	116±66	<0.0001
hsCRP (*mg*/*l*)	3.2±3.1	3.1±3.0	3.3±3.2	0.36
IMT (mm)	0.75±0.2	0.77±0.2	0.72±0.1	0.001
HOMA	3.0±2.1	3.3±2.3	2.8±1.9	0.001
Liver IR index	2.93±0.37	2.95±0.37	2.92±0.37	0.28
OGIS (ml×min^−1^×m^−2^)	381±75	263±64	298±80	<0.0001
Matsuda	73±46	67±43	79±48	<0.0001
Matsuda2	71±45	65±43	77±47	<0.0001
Stumvoll ISI_OGTT_ (µmol×kg^−1^×min^−1^×pM^−1^)	0.96±0.02	0.95±0.02	0.97±0.02	0.29
NGT,IFG, IGT, IFG/IGT n (%)	459/150/118/120(54.2/17.7/13.9/14.2)	189/89/56/80(45.7/21.5/13.5/19.3)	270/61/62/40(62.4/14.1/14.3/9.2	<0.0001

Data are means ± SD. *P* values for differences of continuous variables between the two groups using unpaired Student’s t. Categorical variables were compared by χ^2^ test. M = male; F = female; BMI = body mass index; SBP = systolic blood pressure; DBP = diastolic blood pressure; HDL = high density lipoprotein; hsCRP = high sensitivity C reactive protein; IMT = intima-media thickness; HOMA = homeostasis model assessment; OGIS = oral glucose insulin sensitivity; ISI = Insulin sensitivity index; Matsuda2 = not including fasting glucose and insulin levels in mean values of glucose and insulin during OGTT; IFG = impaired fasting glucose; IGT = impaired glucose tolerance.

Age and gender adjusted univariate correlations between anthropometric and metabolic variables and IMT in the whole study group are showed in Table 2. IMT was significantly correlated with BMI, waist circumference, fat mass, systolic blood pressure (SBP), diastolic blood pressure (DBP), triglycerides, HDL cholesterol, hsCRP, fasting glucose, 2-h glucose, fasting insulin, and 2-h insulin. Among these variables, BMI exhibited the strongest association (r = 0.23). The Stumvoll ISI_OGTT_ index was correlated with IMT more strongly than the other indexes of IR. The same degree of correlation was observed with the canonical Matsuda index and with the Matsuda2 index which does not average fasting glucose and insulin levels when computing mean values of glucose and insulin during OGTT.

**Table 2:** Age and gender adjusted univariate correlations between anthropometric and metabolic variables and IMT in the whole study group.

Variables	IMT	
	Pearson’s correlation coefficient (*r*)	*P*
BMI *(kg/m^2^)*	0.23	<0.0001
Waist circumference (c*m*)	0.16	<0.0001
Fat mass (*%*)	0.18	<0.0001
SBP *(mmHg)*	0.13	<0.0001
DBP *(mmHg)*	0.08	0.02
Fasting Glucose (*mgl/dl*)	0.11	0.002
90-min Glucose (*mgl/dl*)	0.14	0.001
2-h Glucose (*mgl/dl*)	0.09	0.01
Fasting Insulin *(µU/ml)*	0.14	<0.0001
2-h Insulin *(µU/ml)*	0.12	0.001
Total cholesterol (*mg/dl*)	0.03	0.41
HDL (*mg/dl*)	−0.10	0.008
Triglycerides (*mg/dl*)	0.05	0.13
hsCRP (*mg*/*l*)	0.12	0.001
HOMA	0.14	<0.0001
Liver IR index	0.18	<0.0001
OGIS (ml×min^−1^×m^−2^)	−0.17	<0.0001
Matsuda	−0.14	<0.0001
Matsuda2	−0.14	<0.0001
Stumvoll ISI_OGTT_ (µmol×kg^−1^×min^−1^×pM^−1^)	−0.25	<0.0001

*P* values refer to results after analysis with adjustment for age and gender. IMT = intima-media thickness; HOMA = homeostasis model assessment; OGIS = oral glucose insulin sensitivity; Matsuda2 = not including fasting glucose and insulin levels in mean values of glucose and insulin during OGTT; ISI = Insulin sensitivity index.

Age and gender adjusted univariate correlations between IR indexes and cardiovascular risk factors in the whole study group are showed in Table 3. The IR indexes correlated significantly (*P*<0.0001) with all cardiovascular risk factors examined (Table 3). The Stumvoll ISI_OGTT_ index was correlated with waist circumference and hsCRP more strongly than the other indexes of IR (Table 3).

**Table 3:** Age and gender adjusted univariate correlations between insulin resistance indexes and cardiovascular risk factors in the whole study group.

	HOMA		Liver IR index		OGIS		Matsuda		Stumvoll ISI_OGTT_	
	Pearson’s correlation coefficient (*r*)	*P*	Pearson’s correlation coefficient (*r*)	*P*	Pearson’s correlation coefficient (*r*)	*P*	Pearson’s correlation coefficient (*r*)	*P*	Pearson’s correlation coefficient (*r*)	*P*
Waist circumference (c*m*)	0.40	<0.0001	0.63	<0.0001	−0.41	<0.0001	−0.42	<0.0001	−0.75	<0.0001
SBP *(mmHg)*	0.11	0.001	0.20	<0.0001	−0.15	<0.0001	−0.17	<0.0001	−0.25	<0.0001
DBP *(mmHg)*	0.13	<0.0001	0.20	<0.0001	−0.18	<0.0001	−0.16	<0.0001	−0.23	<0.0001
total cholesterol(*mg/dl*)	0.01	0.99	0.03	0.40	−0.12	<0.0001	−0.07	0.03	−0.01	0.77
HDL (*mg/dl*)	−0.26	<0.0001	−0.45	<0.0001	0.20	<0.0001	0.24	<0.0001	0.24	<0.0001
Triglycerides (*mg/dl*)	0.19	<0.0001	0.27	<0.0001	−0.18	<0.0001	−0.21	<0.0001	−0.19	<0.0001
hsCRP (*mg*/*l*)	0.26	<0.0001	0.27	<0.0001	−0.22	<0.0001	−0.24	<0.0001	−0.34	<0.0001

*P* values refer to results after analysis with adjustment for age and gender. SBP = systolic blood pressure; DBP = diastolic blood pressure; HDL = high density lipoprotein hsCRP = high sensitivity C reactive protein; HOMA = homeostasis model assessment; OGIS = oral glucose insulin sensitivity; ISI = Insulin sensitivity index; OGTT = oral glucose tolerance test.

To estimate the independent contribution of indexes of IR to carotid IMT, we carried out a multiple regression analysis using carotid IMT as the dependent variable and including age, gender, BMI, SBP, DBP, diastolic blood pressure, triglycerides, total cholesterol, HDL cholesterol, hsCRP and indexes of IR as independent variables. As shown in Table 4, all indexes of IR remained significantly associated with carotid IMT with the Stumvoll ISI_OGTT_ index showing the strongest independent association. When BMI was entered into the regression model 1, the four indexes of IR that remained significantly associated with carotid IMT were Matsuda, OGIS, Liver IR, and Stumvoll ISI_OGTT_ with the latter index showing the strongest independent association (Table 5).

**Table 4:** Multiple regression analysis testing the independent association between indexes of insulin resistance and carotid intima–media thickness (IMT) in a model including age, gender, systolic blood pressure, diastolic blood pressure, triglycerides, total cholesterol, HDL cholesterol, and hsCRP as independent variables (Model 1).

HOMA		Liver IR index		OGIS		Matsuda		Stumvoll ISI_OGTT_	
β coefficient	*P*	β coefficient	*P*	β coefficient	*P*	β coefficient	*P*	β coefficient	*P*
0.13	<0.0001	0.15	<0.0001	−0.17	<0.0001	−0.13	<0.0001	−0.22	<0.0001

HOMA = homeostasis model assessment; OGIS = oral glucose insulin sensitivity; ISI = Insulin sensitivity index.

**Table 5:** Multiple regression analysis testing the independent association between indexes of insulin resistance and carotid intima–media thickness (IMT) in a model including age, gender, BMI systolic blood pressure, diastolic blood pressure, triglycerides, total cholesterol, HDL cholesterol, and hsCRP as independent variables (Model 2).

HOMA		Liver IR index		OGIS		Matsuda		Stumvoll ISI_OGTT_	
β coefficient	*P*	β coefficient	*P*	β coefficient	*P*	β coefficient	*P*	β coefficient	*P*
0.07	0.04	0.04	0.38	−0.13	0.001	−0.08	0.03	−0.21	0.001

HOMA = homeostasis model assessment; OGIS = oral glucose insulin sensitivity; ISI = Insulin sensitivity index.

**Figure 1:**
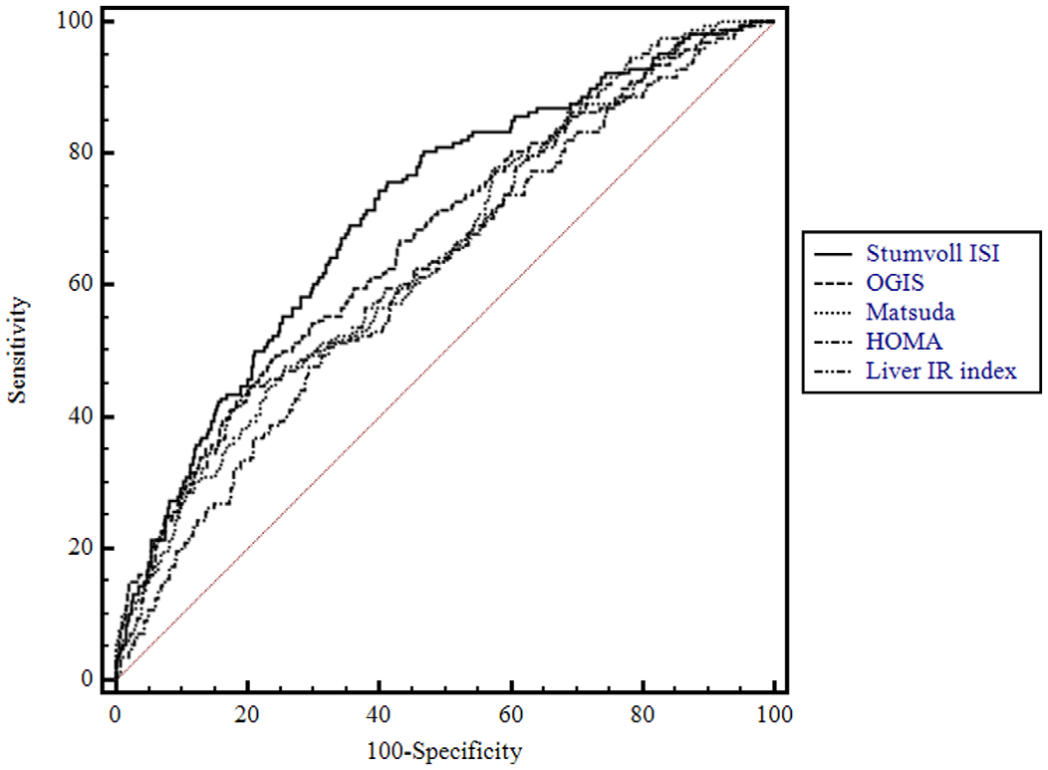
ROC curve analyses for detecting subjects with early atherosclerosis according to HOMA, Matsuda, OGIS, Stumvoll ISI_OGTT_, and Liver IR indexes.

The area under the ROC curve (AUC) was used to evaluate the accuracy of the IR indexes in identifying individuals with vascular damage, defined as IMT >0.9 mm. The AUC for the Stumvoll ISI_OGTT_ index was significantly higher (0.710) as compared with the AUCs of Matsuda (0.642, *P* = 0.0009), OGIS (0.666, *P* = 0.04), HOMA (0.611, *P*<0.0001) and Liver IR (0.648, *P* = 0.0008) indexes (Figure 1).

Next, the subjects were stratified into tertiles according to the values of the IR indexes. In a logistic regression model adjusted for age and gender, SBP, DBP, triglycerides, total cholesterol, HDL cholesterol, hsCRP, subjects in the lowest tertile of the Stumvoll ISI_OGTT_ index had a 3.71-fold higher risk of having vascular damage as compared with those in the highest tertile (OR 3.71, 95% CI 2.07–6.61), subjects in the lowest tertile of Matsuda index had a 1.87-fold higher risk of having vascular damage as compared with those in the highest tertile (OR 1.87, 95% CI 1.09–3.21), subjects in the lowest tertile of OGIS index had a 2.21-fold higher risk of having vascular damage as compared with those in the highest tertile (OR 2.21, 95% CI 1.25–3.93), subjects in the highest tertile of HOMA index had a 2.05-fold higher risk of having vascular damage as compared with those in the lowest tertile (OR 2.05, 95% CI 1.20–3.51), and subjects in the highest tertile of Liver IR index had a 2.45-fold higher risk of having vascular damage as compared with those in the lowest tertile (OR 2.45, 95% CI 1.40–4.31).

## Discussion

IR is typically defined as a condition of diminished ability of target tissues to take up and metabolize glucose in response to insulin, and it plays a major role in the development of type 2 diabetes. IR is also a key feature of a spectrum of metabolic abnormalities such as obesity and glucose intolerance, and is associated with multiple cardiovascular risk factors (visceral adiposity, hypertension, atherogenic dyslipidemia), a clinical constellation that has been referred to as metabolic syndrome [4]. IR is a predictor of atherosclerotic cardiovascular disease [2], and this association has vast social implications, and calls for intensive investigation of the causes of the disease, to optimize its treatment and to possibly prevent its onset.

IR occurs in multiple tissues, particularly in the skeletal and cardiac muscle, adipose tissue and liver [23]. Although accurate methods have been developed to measure directly tissue-specific IR with the glucose clamp technique combined with tracers, these methods are impractical for large scale epidemiological studies since they are invasive, laborious, costly, and time-consuming. Therefore, simpler methods to measure IR have been developed using fasting insulin and/or glucose levels alone [13] or in combination with insulin and glucose levels during an OGTT [14]–[17], a widely employed test to assess glucose homeostasis in clinical practice. All surrogates indexes correlate reasonably well with insulin sensitivity measured by euglycemic-hyperinsulinemic clamp [13]–[17], although they differentially reflect the contribution of individual organs, e.g., liver and muscle, to insulin resistance. Indexes derived from measurements of fasting plasma glucose and insulin levels such HOMA [13] primarily reflect the basal measurement of hepatic glucose production (HGP) [24], while the liver IR index was specifically developed to measure hepatic IR [17]. Finally, the Matsuda and the Stumvoll ISI_OGTT_ indexes primarily reflect muscle insulin sensitivity [14], [15], [24], whereas the OGIS reflects the metabolic clearance rate of glucose [16].

In the present study, we documented that these IR indexes are differentially associated with vascular damage, as assessed by IMT of common carotid artery [18]. We found that the Stumvoll ISI_OGTT_ index correlates better with IMT than the four other surrogate indexes as indicated by the significantly higher value of the area under the ROC curve and by the increased risk of having vascular damage for subjects in the lowest tertile of the Stumvoll ISI_OGTT_ index as compared to the corresponding tertiles of the other surrogate indexes. To the best of our knowledge, this is the first study to investigate the associations between different surrogate indexes of IR and IMT in a large cohort of non-diabetic individuals.

The better association observed between the Stumvoll ISI_OGTT_ index and vascular damage may be partially explained by the fact that it takes into consideration BMI in addition to insulin and glucose concentrations during an OGTT. Consequently, this index may signal not only muscle IR but also other important domains for vascular damage such a visceral adiposity, and low-grade inflammation. In support of this notion, we found that, compared to the other surrogate indexes of IR, the Stumvoll ISI_OGTT_ index was more strongly correlated with waist circumference, and hsCRP circulating levels. Several adipokines released from adipocytes and adipose tissue-derived macrophages are risk factors for cardiovascular disease [25]. These adipokines can contribute to inflammation and to the increased synthesis of inflammatory proteins, such as CRP. CRP in turn may directly affect vasculature facilitating the development of atherosclerosis, and, eventually, cardiovascular diseases [26]. However, we cannot exclude that other variables employed in the Stumvoll ISI_OGTT_ index such as Insulin_120_ and Glucose_90,_ which exhibited a significant association with carotid IMT, may account for the present results.

The strengths of the present study are the relatively large sample size, the homogeneous population, the carefully characterized phenotype of individuals with different degree of glucose tolerance, and the exclusion of confounding conditions such as previous cardiovascular disease, history of malignant disease, and liver or kidney failure. Nevertheless, the present study has some limitations. The cross-sectional nature of our study does not provide insight into the time course of atherosclerotic cardiovascular disease, and, therefore, no conclusions regarding cause-effect relationships can be made. In addition, the present findings are only based on Caucasian individuals, and results might vary as a function of ethnic group. Indeed, previous studies have shown differences in insulin sensitivity among different ethnic groups with Mexican Americans and African Americans being more insulin resistant than Caucasians [27], [28], likely due to sociodemographic, lifestyle, anthropometric, and genetic characteristics. Finally, the study included only non-diabetic subjects, thus excluding from the analysis subjects at very high risk of cardiovascular disease such as patients with type 2 diabetes.

### Conclusions

In conclusion, in the present study we demonstrated that, among the different validated surrogates indexes of IR, the Stumvoll ISI_OGTT_ index correlates better with vascular damage, as assessed by IMT of common carotid artery, in a large cohort of non-diabetic individuals. Thus, the Stumvoll ISI_OGTT_ index might be a significant indicator of vascular damage with an important clinical significance.
